# Factors influencing image quality in Tibetan children by optical coherence tomography

**DOI:** 10.3389/fmed.2025.1495527

**Published:** 2025-01-17

**Authors:** Yao Yao, Jiawen Liu, Lei Li, Weiwei Chen, Zhaojun Meng, Jing Fu

**Affiliations:** ^1^Beijing Key Laboratory of Ophthalmology and Visual Sciences, Beijing Tongren Eye Center, Beijing Tongren Hospital, Capital Medical University, Beijing, China; ^2^Wilmer Eye Institute, Johns Hopkins Medicine, Johns Hopkins University, Baltimore, MD, United States

**Keywords:** optical coherence tomography, image quality, children, Tibetan, clinical reliability

## Abstract

**Purpose:**

This study aim to investigate the clinical findings of subjects characteristics and image quality related factors in Tibetan children by optical coherence tomography (OCT) in epidemiological cohort study.

**Methods:**

Participants were 1,856 first-grade students (mean age = 6.82 ± 0.46 years) from seven selected elementary schools in Lhasa. Following comprehensive systemic and ophthalmic examinations, OCT scans were assessed by specialists with manual segmentation as needed.

**Results:**

A total of 1,698 students completed the examination protocol in this study (91.5%). After manual screening, 1,447 (78%) and 1,289 (70%) images could be analyzed in the macular and optic disc regions, respectively. Common image flaws were blinking or fixation error (70%+), poor focusing, and positioning errors. Among students who have completed OCT, a higher percentage of boys (*X*^2^ = 8.48, *P* = 0.004) and suburban students (*X*^2^ = 34.97, *P* < 0.001) with younger age (*t* = -2.20, *P* = 0.03), worse near vision (*t* = -3.95, *P* < 0.001), higher IOP (*t* = 2.38, *P* = 0.017) and higher heart rate (*t* = 3.15, *P* = 0.002) have unsatisfactory image quality in the macular region, almost same as the optic disc region. Students in suburban schools (OR = 1.74, *P* < 0.001) with lower near VA (OR = 6.64, *P* < 0.001) or boys (OR = 0.78, *P* = 0.03) were more likely to have worse image quality on OCT scans when corrected for ethnicity. Manual segmentation was more prevalent in the optic disc region, resulting in increased retinal thickness across most subregions.

**Conclusion:**

This study underscores the imperative for stringent image quality control in pediatric OCT assessments to ensure precise clinical outcomes.

## Introduction

Optical coherence tomography (OCT) constitutes a non-invasive and well-tolerated imaging modality leveraging low coherence light reflected by ocular tissues to generate high-resolution images (1). This technique empowers clinicians to produce three-dimensional (3D) renderings of intraocular structures *in vivo* (2, 3). Beyond subjective qualitative assessment, these images lend themselves to objective analysis, facilitating quantitative measurements such as stratified retinal thickness across distinct retinal zones (4, 5). With advancements in image resolution, quality, and software capabilities, the quantitative scrutiny of OCT images has undergone significant refinement (6, 7). The faster scanning speed and higher imaging quality allow it to be performed also in patients with poor cooperation (e.g., children) (8–10). Although the accuracy and reproducibility of OCT applications in pediatric contexts have undergone scrutiny, empirical evidence validating its clinical utility across diverse demographics remains lacking (11).

Precise and highly reproducible quantitative measurements serve as indispensable diagnostic and monitoring tools, guiding optimal treatment decisions across routine medical care and clinical trials alike. However, the variance in measurement values among different OCT instruments underscores the need for standardized algorithms for image recognition and segmentation (12). The application of OCT in pediatric cohorts introduces additional intricacies, owing to factors such as limited patient cooperation, diminutive ocular dimensions, and the imperative of precise image alignment and interpretation (11, 13). Despite these hurdles, OCT holds immense promise as a non-invasive, objective tool for delineating ocular morphology and pathology in children. A nuanced understanding of the determinants of successful OCT imaging and the factors shaping image quality is imperative for refining screening protocols, augmenting diagnostic precision, and guiding public health initiatives geared toward pediatric ocular wellness.

The Lhasa Childhood Eye Study (LCES) represents a pioneering effort, aiming to investigate the clinical findings of subject characteristics and factors influencing image quality in Tibetan children using OCT within an epidemiological cohort setting. The unique demographic and environmental characteristics of the Tibetan population, coupled with the challenges inherent in conducting large-scale pediatric ocular screenings in resource-limited settings, underscore the significance of this study. Through a comprehensive approach encompassing clinical examination, OCT imaging, and systemic evaluation, this study seeks to elucidate the epidemiology of ocular conditions, identify risk factors for poor image quality in OCT, and inform strategies for improving the efficiency and effectiveness of pediatric ocular screening programs.

## Materials and methods

### Study design and population

The LCES constituted a school-based, observational cohort epidemiological inquiry into childhood ocular ailments (14). The study protocol adhered to the principles of the Declaration of Helsinki and obtained approval from the Ethics Committee of the Beijing Tongren Hospital, Capital Medical University (No. TRECKY2019-058). Written informed consent was provided by all participants’ parents or legal guardians. The LCES employed stratified cluster sampling among grade one students in Lhasa, Tibet, located in the southwest of China. Following local government assessment, 27 out of 28 elementary schools effectively participating in the LCES were stratified into three tiers. Ultimately, seven elementary schools were randomly selected as samples, encompassing 1,942 grade 1 students through stratified cluster sampling. The study commenced in 2019 and will continue for an additional 5 years until the students transition to junior high school.

### Study protocols and examination methods

All participants underwent comprehensive ophthalmic examinations, encompassing evaluations of uncorrected and best-corrected visual acuity (BCVA), near visual acuity (VA), stereopsis acuity (S0001, STEREO, USA), ocular dominance, slit-lamp biomicroscopy assessment (SL-3G, Topcon, Tokyo, Japan), non-contact tonometry (CT-800, Topcon, Tokyo, Japan), ocular alignment, and objective refraction measurement before and after cycloplegia, in addition to OCT and retinal photography.

Anthropometric parameters such as body height and weight, blood oxygen saturation, and heart rate were also assessed. Body mass index (BMI), calculated as the ratio of body weight (kg) divided by the square of body height (m), served as a measure of body composition. Oxygen saturation and heart rate were measured twice using a digital fingertip pulse oximeter, with the average value utilized for statistical analysis.

### OCT measurements

**FIGURE 1:**
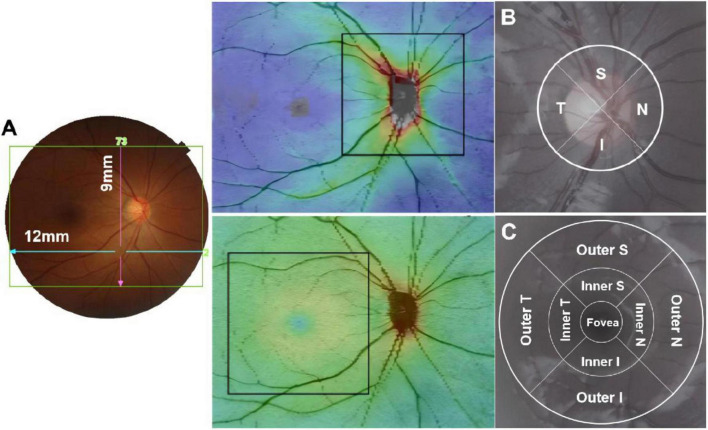
The 12 mm × 9 mm scan region **(A)**; Measurement areas for circumpapillary circle **(B)**; macular grid **(C)** overlaid with example projected images. T, temporal; S, superior; N, nasal; I, inferior.

Optical coherence tomography examination was conducted by a certified and experienced technician utilizing spectral-domain OCT (3D OCT-1, Topcon, Tokyo, Japan). The Topcon 3D OCT-1 is a non-contact SD-OCT system renowned for its capability to perform a fully automated “alignment, focus, and capture” procedure, capturing an impressive 50,000 axial scans per second (15, 16). In this study, we opted for the “3D wide scan mode,” which entails a 12 mm × 9 mm scan (Figure 1A), providing detailed measurement and topographical maps of the optic nerve and macula (Figures 1B, C). Recognizing the influence of circadian rhythms and cycloplegia on choroidal thickness, all examinations were conducted prior to cycloplegia, specifically between 1 p.m. and 4 p.m.

To acquire optic nerve head parameters, a 3.4 mm diameter temporal-superior-nasal-inferior-temporal (TSNIT) circle was automatically positioned at the center of the optic disc. Circumpapillary retinal nerve fiber layer (cpRNFL) thickness was evaluated across 4 quadrants and 12 clock-hour sectors around the TSNIT circle (Figure 1B). Macular full retinal thickness (FRT) was determined as the distance between the inner limiting membrane (ILM) and the boundary of the outer segments/retinal pigment epithelium, delineated according to the Early Treatment Diabetic Retinopathy Study (ETDRS) map, with its nine quadrants illustrated in Figure 1C. The diameters of the three circles on the ETDRS map were 1, 3, and 6 mm.

In this study, image quality was categorized as “Qualified,” “Acceptable,” and “Unqualified” in accordance with the OSCAR-IB consensus criteria for retinal OCT quality assessment (15). Qualified images met criteria such as signal strength exceeding 6 (out of a maximum of 10), centered image scanning position, and absence of other quality issues. Unqualified images exhibited significant exposure abnormalities, refractive interstitial turbidities, extensive contamination, missing information, or irrelevant images. Acceptable images presented minor exposure issues, limited contaminations with negligible impact on interpretation, slight out-of-focus or blurred characteristics, among others. In cases of automatic segmentation errors related to foveal centration, boundary segmentations, or disc margin in fast maps, manual segmentation was executed based on anatomical considerations (11, 17). Foveal modification involved the relocation of the ETDRS and Macula 6 grid center location, while adjustments to the disc margin and center corresponded to alterations in the disc outline and location, respectively. Other modifications encompassed segmentation adjustments between boundaries (16). Evaluation of all collected images was performed by Yao Yao, with clinical data and medical records withheld during the grading process. Image enhancement tools were not utilized. In instances of ambiguity, a panel of ophthalmologists (Jing Fu and Zhaojun Meng) reassessed the OCT images to reach a consensus. All manual segmentation was electronically documented, and images from the right eye were utilized for analysis in the present study.

### Statistical analysis

Values were presented as mean ± standard deviation (SD) for continuous variables and as percentage for categorical variables. All data was analyzed using Statistical Analysis System software (version 9.4, SAS Inc., Cary, NC, USA). Independent samples *t*-tests were utilized to compare the differences in systemic and ocular influencing factors among different OCT completion status groups. Chi-square tests were employed to assess differences in gender, school location, ethnicity, and amblyopia among groups. Multivariate logistic analysis was conducted to evaluate the influence of various related factors on OCT image completion and quality. Paired *t*-tests were employed to compare differences in quantitative measurement values of OCT images before and after manual adjustments. A two-tailed *P*-value of less than 0.05 was defined as statistically significant at the 95% confidence interval level.

## Results

### Study population and general characteristics

A total of 1,856 subjects were enrolled in the first year of LCES, with a mean age of 6.82 ± 0.46 years. Notably, 53% of the included students were male, while 94% were Tibetan. Demographic data of the cohort are shown in Table 1. There were 150 participants who failed to complete protocols or examine procedures and 8 had ocular diseases who could not complete the OCT scans, giving an analyzable fraction of 91.5%. The average abxge of students performed OCT assessment was 6.83 ± 0.46 years, 53.02% of the participants were boy, 94.93% were Tibetan minority.

**TABLE 1:** Systemic parameters of the study population.

Parameters	Mean	SD
Visual acuity	0.11	0.19
Near visual acuity	−0.16	0.14
Intraocular pressure, mmHg	16.02	2.73
Height, cm	120.55	5.52
Weight, kg	22.96	3.69
Body mass index, kg/m^2^	15.74	1.80
Heart rate	95.27	14.27
Systolic blood pressure, mmHg	95.67	7.99
Diastolic blood pressure, mmHg	64.68	6.57
Mean arterial blood pressure, mmHg	85.34	6.65
Blood oxygen saturation, %	92.68	3.07
Spherical equivalent, D	1.07	0.92
Best-corrected visual acuity	0.03	0.10
Age	6.82	0.46

### Image enrollment and screening

**FIGURE 2:**
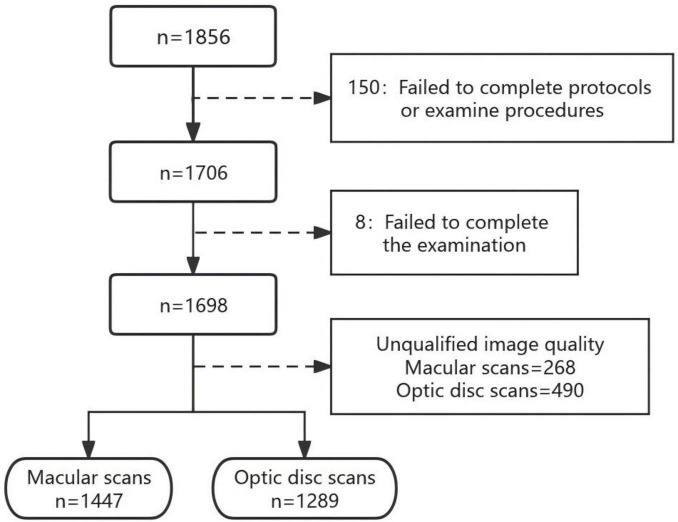
Inclusion and exclusion of study participants.

After manual segmentation, another 268 scans in the macula area and 490 scans in the optic disc area which did not meet the image criteria were excluded, respectively. We were ultimately left with 1,447 macular scans (78%) and 1,289 (70%) optic disc scans. The enrollment and screening process for this study is shown in Figure 2. Of the failed images, the most common cause was scanned image defects due to blinking or fixation error (over 70%), followed by image blurring due to poor focusing and uncorrectable image positioning errors.

### Retinal thickness and distribution of macula and cpRNFL

Table 2 showed the thickness and distribution of the macula and cpRNFL before and after the manual adjustment. The average retinal thickness of macula was 278.57 ± 13.40 μm before the manual segmentation and 279.08 ± 11.06 μm after that. In the inner circle of macular full retina, the superior region is the thickest area, followed by the inferior, nasal and temporal regions. In the outer ring, the nasal region was the thickest, followed by the superior, inferior and temporal regions. While the average thickness of cpRNFL was 101.58 ± 27.20 and 112.39 ± 12.31 μm before and after the adjustment, respectively. The thickest area was the inferior region, followed by the superior, the temporal and the nasal quadrants. The cpRNFL thickness in the 12 sections is more discrete than the 4 sections.

**TABLE 2:** Optical coherence tomography parameters before and after the manual segmentation.

	Mean	SD		Mean	SD
Macular (1,698), mm			After adjustment (1,447)		
Average	278.57	13.40	Average	279.08	11.06
Foveal minimum	171.90	29.00	Foveal minimum	168.44	16.80
Center	205.24	25.90	Center	201.75	18.14
Inner temporal	287.71	22.85	Inner temporal	289.48	13.31
Inner superior	304.49	16.43	Inner superior	305.50	12.66
Inner nasal	294.12	20.72	Inner nasal	295.70	16.55
Inner inferior	300.89	17.09	Inner inferior	301.74	12.62
Outer temporal	264.57	27.05	Outer temporal	265.46	14.91
Outer superior	276.34	14.20	Outer superior	277.12	12.27
Outer nasal	295.25	17.45	Outer nasal	295.63	13.57
Outer inferior	267.04	14.96	Outer inferior	266.88	12.13
Total volume (mm^3^)	7.87	0.38	Total volume (mm^3^)	7.89	0.31
Optic disc (1,698), mm			After adjustment (1,289)		
Global average	101.58	27.20	Global average	112.39	13.31
Temporal	70.39	25.08	Temporal	79.60	13.80
Superior	130.07	37.05	Superior	144.25	19.40
Nasal	76.01	25.95	Nasal	79.24	16.46
Inferior	129.65	40.90	Inferior	146.34	20.58
9 o’clock	56.13	20.55	9 o’clock	63.08	12.03
10 o’clock	82.98	30.24	10 o’clock	94.06	18.00
11 o’clock	131.25	45.71	11 o’clock	149.58	25.51
12 o’clock	138.34	43.71	12 o’clock	152.56	26.98
1 o’clock	120.57	37.47	1 o’clock	130.60	24.42
2 o’clock	93.95	32.36	2 o’clock	99.45	22.83
3 o’clock	60.35	24.14	3 o’clock	61.45	15.07
4 o’clock	73.57	30.13	4 o’clock	76.82	20.56
5 o’clock	115.16	40.41	5 o’clock	127.82	25.69
6 o’clock	143.95	49.67	6 o’clock	161.35	29.35
7 o’clock	130.16	48.31	7 o’clock	149.84	27.11
8 o’clock	72.09	29.38	8 o’clock	81.65	17.66

### Multivariate analysis of parameters associated with image quality

We compared general systemic and ophthalmic factors in subjects who passed and failed the image quality check. Among students who have completed OCT, a higher percentage of boys (*X*^2^ = 8.48, *P* = 0.004) and suburban students (*X*^2^ = 34.97, *P* < 0.001) have unsatisfactory image quality. In addition, students with scanned images of poor quality exhibited younger age (*t* = −2.20, *P* = 0.03), worse near vision (*t* = −3.95, *P* < 0.001), higher IOP (*t* = 2.38, *P* = 0.017) and higher heart rate (*t* = 3.15, *P* = 0.002) in the macular region, almost same as the optic disc region (Supplementary Table 1). Multivariate logistic regression analysis showed that students in suburban schools (OR = 1.74, *P* < 0.001) with lower near VA (OR = 6.64, *P* < 0.001) or boys (OR = 0.78, *P* = 0.03) were more likely to have worse image quality on OCT scans when corrected for ethnicity (Table 3).

**TABLE 3:** Multivariate logistic regression analysis under different groups of OCT examination.

Macular	Unstandardized coefficient	*P-*value	Odds ratio	95% confidence interval
**Whether OCT was completed**				
Gender	−0.379	0.031	0.685	0.485, 0.966
School location	−1.068	<0.001	0.344	0.19, 0.622
Amblyopia	−0.405	0.575	0.667	0.162, 2.747
Near visual acuity	1.451	0.013	4.266	1.353, 13.447
Height	0.003	0.887	1.003	0.961, 1.047
Weight	−0.056	0.103	0.946	0.884, 1.011
Visual acuity	0.214	0.725	1.239	0.376, 4.089
Best-corrected visual acuity	1.926	0.129	6.863	0.573, 82.277
**Whether OCT was deleted**				
Gender	−0.392	0.005	0.676	0.515, 0.888
School location	0.641	<0.001	1.899	1.351, 2.669
Near visual acuity	0.615	0.25	1.849	0.649, 5.27
Best-corrected visual acuity	0.019	0.981	1.019	0.224, 4.638
Intraocular pressure	−0.052	0.037	0.949	0.904, 0.997
Heart rate	−0.012	0.025	0.989	0.979, 0.999
Blood oxygen saturation	−0.02	0.367	0.98	0.939, 1.024
Age	0.106	0.51	1.111	0.812, 1.521
**Whether OCT was adjusted**				
School location	1.055	<0.001	2.872	1.616, 5.102
Heart rate	0.023	0.015	1.023	1.004, 1.042
**Optic disc**				
**Whether OCT was completed**				
Near visual acuity	1.576	0.009	4.833	1.491, 15.664
Heart rate	0.004	0.494	1.004	0.992, 1.017
School location	−1.024	0.001	0.359	0.196, 0.657
Gender	−0.367	0.037	0.693	0.491, 0.978
Amblyopia	−0.419	0.56	0.658	0.161, 2.691
Visual acuity	0.197	0.746	1.218	0.37, 4.002
Height	0.001	0.966	1.001	0.959, 1.045
Weight	−0.053	0.124	0.949	0.887, 1.015
Best-corrected visual acuity	1.964	0.107	7.126	0.656, 77.38
**Whether OCT was deleted**				
Near visual acuity	1.893	<0.001	6.64	2.832, 15.57
Heart rate	0.001	0.774	1.001	0.993, 1.01
School location	0.554	<0.001	1.741	1.323, 2.29
Gender	−0.248	0.033	0.78	0.621, 0.98
Ethnicity	−0.497	0.141	0.608	0.314, 1.178
**Whether OCT was adjusted**				
Near visual acuity	2.664	<0.001	14.353	5.431, 37.933
Intraocular pressure	−0.041	0.057	0.96	0.921, 1.001
BMI	−0.071	0.028	0.932	0.875, 0.992
Heart rate	0.004	0.422	1.004	0.995, 1.012
Age	0.11	0.432	1.116	0.849, 1.466
School location	1.148	<0.001	3.153	2.183, 4.554

### Correlation analysis after manual segmentation of images

Except for the images that were directly deleted due to poor quality, some of the images had deviations in position or range alignment, so we restored the proper position of these scans by manual segmentation and compared the image parameters before and after the changes. A significantly higher percentage of 51.4% images in the optic disc area require manual segmentation compared to the macula (4.6%). The OCT scans of suburban students requires more manual segmentation in both the macula and optic disc regions than students in urban schools. Scans of the optic disc area showed that students requiring image adjustments had significantly worse near vision (*t* = 6.84, *P* < 0.001), lower IOP (*t* = −2.19, *P* = 0.03), BMI (*t* = −2.44, *P* = 0.02) and heart rate (*t* = −3.02, *P* = 0.003) and were older (*t* = 4.09, *P* < 0.001) (Supplementary Table 1). The results of the multivariate logistic regression analysis in the optic disc area were consistent with the above. No other significant differences were found in the macular area scans (Table 3).

**FIGURE 3:**
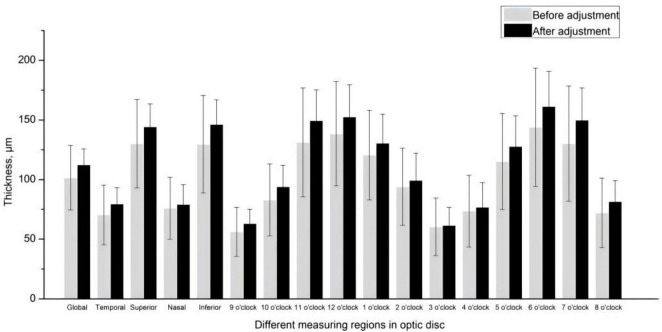
Circumpapillary retinal nerve fiber layer thickness in 4 and 12 subregions before and after the manual segmentation.

Using the values obtained by automatic machine segmentation and measurement, we compared the retinal thickness and optic disc related parameters in each region of the macula and optic disc before and after manual segmentation. We found that adjusted retinal thickness in the macula varied only within a few ETDRS subregions, but there were no significant changes in mean retinal thickness and volume across the region. In contrast, after manual segmentation, significant differences were observed in almost all subregions of the optic disc area, both under 4 and 12 subregions mode (Figure 3). The retinal thickness in most subregions tended to increase after adjustment (Table 4).

**TABLE 4:** Paired *t*-test of OCT measurements before and after manual segmentation.

	Mean	SD	*t*	*P*-value
**Macula**				
Average	−0.03	1.22	−0.87	0.387
Foveal minimum	0.38	9.69	1.49	0.137
Center	0.39	6.70	2.21	0.028
Inner temporal	−0.08	5.09	−0.61	0.542
Inner superior	−0.10	1.91	−2.05	0.041
Inner nasal	−0.19	2.41	−3.02	0.003
Inner inferior	−0.13	2.25	−2.23	0.026
Outer temporal	0.00	2.54	−0.04	0.966
Outer superior	−0.12	1.80	−2.44	0.015
Outer nasal	−0.02	1.39	−0.59	0.555
Outer inferior	0.08	2.16	1.39	0.166
Total volume (mm^3^)	0.00	0.03	−0.92	0.358
**Optic disc**				
Global average	−1.89	10.73	−6.34	<0.001
Temporal	−1.85	10.31	−6.45	<0.001
Superior	−2.62	16.25	−5.78	<0.001
Nasal	0.61	8.81	2.48	0.013
Inferior	−3.66	18.00	−7.29	<0.001
9 o’clock	−1.38	8.81	−5.64	<0.001
10 o’clock	−2.36	12.55	−6.76	<0.001
11 o’clock	−4.22	19.50	−7.76	<0.001
12 o’clock	−2.65	20.48	−4.64	<0.001
1 o’clock	−1.00	13.76	−2.60	0.009
2 o’clock	0.40	11.38	1.25	0.211
3 o’clock	0.65	8.69	2.67	0.008
4 o’clock	0.78	10.07	2.79	0.005
5 o’clock	−1.89	14.61	−4.65	<0.001
6 o’clock	−4.39	23.42	−6.73	<0.001
7 o’clock	−4.60	20.74	−7.97	<0.001
8 o’clock	−1.79	11.80	−5.46	<0.001

## Discussion

Focusing on a large cohort of minority children, this study shed light on the clinical findings of subjects characteristics and image quality related factors in Tibetan children by OCT. In this study, we used manual segmentation to correct errors in automated macular and optic-disc segmentation to better reflect the anatomy represented in each scan. This study found that: (1) high analyzable fraction (91.5%) after exclusion of incomplete protocols or examination procedures; (2) the most significant causes of image failure are image defects and shifts caused by blinking and poor fixation stability in children; (3) multivariate analysis identified suburban residence, lower near VA, and male gender as significant predictors of worse image quality; and (4) differences in retinal thickness and optic disc parameters were observed pre and post-adjustment, with more significant changes in the optic disc area.

This study found that the response rate and coordination of Tibetan children for completing the OCT examination were relatively higher compared to other previous studies. In the Anyang Childhood Eye Study, about 7% of children aged 12 years old had failed images (18). In the Sydney Childhood Eye Study, OCT scans failed in about 21% of children aged 6 years (19). Although our study was conducted among Tibetan children and most 7-year-old Tibetan children are still unable to use Chinese to communicate, we still obtained a pass rate of nearly 85% of the scanned macular area. While this attrition rate is relatively low, it underscores the challenges inherent in longitudinal studies involving young children. Previous studies of screening with the Topcon device have shown an ineligible rate of about 20% and a scan rejection rate of about 2%–5% (16). Also the optic disc has a higher rejection rate than the macula. The primary reasons for image exclusion included defects related to blinking or poor fixation stability, image blurring, and uncorrectable positioning errors. This highlights the importance of rigorous quality control measures in ensuring reliable data acquisition.

In our investigation, a noteworthy correlation emerged between children’s near VA and the quality of OCT scans. Notably, children with superior near VA exhibited higher OCT imaging quality. Healthy children typically demonstrate enhanced ocular accommodation, and the majority of subjects in our study boasted near VA within the normal range. This phenomenon might be attributed to the proximity of internal optic markers of OCT to the eye, whereby myopic acuity can influence children’s fixation on these markers, thereby affecting the speed and quality of OCT scans. Previous research often links fixation stability with anisometropia, high myopia, strabismus, and amblyopia, yet the association with near vision remains largely unexplored (20, 21). Our study’s distribution of near visual acuity largely fell within the normal range compared to past studies, suggesting that a child’s near vision may exert a substantial influence on the quality of OCT examinations, particularly when the fixation optic marker is situated nearby (22, 23). Despite the absence of standardized clinical guidelines for near visual acuity measurement, its significance in determining the rehabilitation of visually impaired children cannot be overstated, underscoring the necessity for further exploration of the association between fixation stability and near visual acuity.

Furthermore, our findings reveal a significant association between the quality of OCT scans and the educational locale and gender of the children. Notably, girls demonstrated significantly higher proficiency in completing OCT scans with qualified images compared to boys, aligning with common perceptions that boys tend to exhibit more rambunctious behavior and are less inclined to cooperate during examinations. Moreover, our study observed that children attending schools in suburban areas underwent more OCT scans necessitating deletion and adjustment. Interestingly, despite exhibiting greater discipline during examinations, children from suburban schools often face language barriers due to their lower proficiency in Chinese, potentially hindering their performance in examinations. This study also revealed that lower image quality is associated with higher intraocular pressure and heart rate. Non-contact tonometry, due to its air-puff mechanism, often yields elevated intraocular pressure readings in children, as blinking or eyelash obstruction commonly interferes with measurements. Similarly, higher heart rates may be linked to the heightened activity levels typical of children. Therefore, increased intraocular pressure and heart rate may indicate that children are more active, sensitive, and less cooperative during examinations, ultimately resulting in reduced image quality.

Recent research underscores the reproducibility and clinical utility of OCT in pediatric populations (24–26). Our study reveals that OCT examinations conducted post-dilation in school-age children, coupled with the software’s automated segmentation calculations, yield informative results. However, automatic positioning segmentation errors contribute to thinner measured retinal thicknesses, consistent with prior investigations (27–29). Literature suggests that erroneous positioning of the optic disc or macular region by OCT often leads to deviations from normal values (30, 31). Topcon’s normal population database highlights the necessity for manual adjustments to rectify automatic segmentation errors, including grid locations, boundary segmentations, and disc margins with Fastmap, executed by clinical experts (16). Such errors may stem from inherent flaws in the machine algorithm. By manually repositioning the measurement region to its correct anatomical location, more accurate measurement values can typically be obtained. Our study also underscores that in the absence of obvious artifacts or image loss, most images can yield relatively valid measurement values through proper repositioning of the measurement area. This underscores the importance of manual supervision and adjustment in epidemiological screening or clinical practice to ensure the accuracy and authenticity of OCT scan results.

This study possesses several strengths as well as limitations. On one hand, the sample size is considerable, the examination protocols are comprehensive, the dataset is comprehensive, and the sample demographics are robust, particularly targeting minority children. However, the investigation solely examined the overall retinal thickness in the macular and optic disc regions, without delving into the perfection of OCT’s layered functionality. Furthermore, there exists a degree of subjectivity in the manual segmentation process of OCT scans. Additionally, the study predominantly included healthy children, warranting further exploration of OCT scanning outcomes in diverse retinal pathologies.

## Conclusion

In conclusion, completion rates of OCT examinations for large-scale pediatric eye screening can reach high levels, and their completion rates are related to educational location, gender, visual acuity, and amblyopia; worse near vision significantly affects OCT image quality and completion rate; OCT scans in pediatric subjects pose greater challenges for alignment in the optic disc area compared to the macular region, and retinal thickness in both regions tends to be thinner than normal in the automated segmentation computations of the OCT apparatus. These discoveries enrich our comprehension of the factors influencing image quality in pediatric OCT imaging and underscore the imperative of rigorous quality assurance measures in large-scale epidemiological investigations.

## Data Availability

The raw data supporting the conclusions of this article will be made available by the authors, without undue reservation.
