# Supplementary material for: Factors influencing image quality in Tibetan children by optical coherence tomography
Source: Front Med (Lausanne). 2025 Jan 17;12:1495527. doi: 10.3389/fmed.2025.1495527 (PMC11782558; doi:10.3389/fmed.2025.1495527)
Supplement: Supplementary file 1 [file Table_1.docx]

**Supplementary Table 1. Comparison of systemic factors under different groups of OCT examination**

|  | whether OCT was completed | | whether OCT was deleted | | whether OCT was adjusted | |
| --- | --- | --- | --- | --- | --- | --- |
| **Macula** | t | P value | t | P value | t | P value |
| Visual acuity | -2.24 | 0.03 | -1.39 | 0.16 | -1.54 | 0.12 |
| Near visual acuity | -2.38 | 0.02 | -3.95 | <0.001 | -0.34 | 0.73 |
| Intraocular pressure | 0.36 | 0.72 | 2.38 | 0.02 | 1.18 | 0.24 |
| Height | 2.06 | 0.04 | 0.69 | 0.49 | 1.20 | 0.23 |
| Weight | 2.09 | 0.04 | -0.06 | 0.96 | 0.63 | 0.53 |
| BMI | 1.35 | 0.18 | -0.35 | 0.73 | 0.19 | 0.85 |
| Heart rate | -1.04 | 0.30 | 3.15 | 0.002 | -1.84 | 0.07 |
| Blood oxygen saturation | 1.38 | 0.17 | 1.67 | 0.10 | -0.88 | 0.38 |
| Spherical equivalent | 0.15 | 0.88 | -1.54 | 0.13 | -0.45 | 0.65 |
| Best-corrected visual acuity | -2.66 | 0.01 | -1.70 | 0.09 | -0.87 | 0.39 |
| Age | 0.60 | 0.55 | -2.20 | 0.03 | -1.39 | 0.16 |
| Image quality |  |  | 9.40 | <0.001 | 0.48 | 0.63 |
|  | X^2^ | P value | X^2^ | P value | X^2^ | P value |
| Gender | 4.16 | 0.04 | 8.48 | 0.004 | 0.02 | 0.90 |
| School location | 13.47 | <0.001 | 34.97 | <0.001 | 9.36 | 0.002 |
| Ethnicity | 3.19 | 0.07 | 1.50 | 0.22 | 0.13 | 0.58 |
| Amblyopia | 4.74 | 0.05 | 0.00 | 0.60 | 2.29 | 0.17 |
| **Optic disc** |  |  |  |  |  |  |
| Visual acuity | -2.24 | 0.03 | 0.11 | 0.91 | 0.73 | 0.47 |
| Near visual acuity | -2.38 | 0.02 | -5.59 | <0.001 | 6.86 | <0.001 |
| Intraocular pressure | 0.36 | 0.72 | 0.40 | 0.69 | -2.19 | 0.03 |
| Height | 2.06 | 0.04 | 0.43 | 0.67 | 1.35 | 0.18 |
| Weight | 2.09 | 0.04 | 0.05 | 0.96 | -1.05 | 0.29 |
| BMI | 1.35 | 0.18 | -0.12 | 0.90 | -2.44 | 0.02 |
| Heart rate | -1.04 | 0.30 | 2.31 | 0.02 | -3.02 | 0.003 |
| Blood oxygen saturation | 1.38 | 0.17 | 0.98 | 0.33 | 0.74 | 0.46 |
| Spherical equivalent | 0.15 | 0.88 | -0.68 | 0.50 | -1.76 | 0.08 |
| Best-corrected visual acuity | -2.44 | 0.02 | -0.58 | 0.56 | -0.33 | 0.74 |
| Age | 0.60 | 0.55 | -0.90 | 0.37 | 4.10 | <0.001 |
| Image quality |  |  | 7.57 | <0.001 | -2.87 | 0.004 |
|  | X^2^ | P value | X^2^ | P value | X^2^ | P value |
| Gender | 4.16 | 0.04 | 32.60 | <0.001 | 0.32 | 0.57 |
| School location | 13.47 | <0.001 | 26.04 | <0.001 | 71.12 | <0.001 |
| ethnicity | 3.19 | 0.07 | 5.36 | 0.02 | 3.32 | 0.07 |
| Amblyopia | 4.74 | 0.05 | 0.85 | 0.40 | 0.46 | 0.50 |
